# Supplementary material for: Heterogeneity in Vaccinal Immunity to SARS-CoV-2 Can Be Addressed by a Personalized Booster Strategy
Source: Vaccines (Basel). 2023 Apr 6;11(4):806. doi: 10.3390/vaccines11040806 (PMC10140995; doi:10.3390/vaccines11040806)
Supplement: Supplementary file 1 [file vaccines-11-00806-s001.zip › vaccines-2235637-supplementary.pdf]

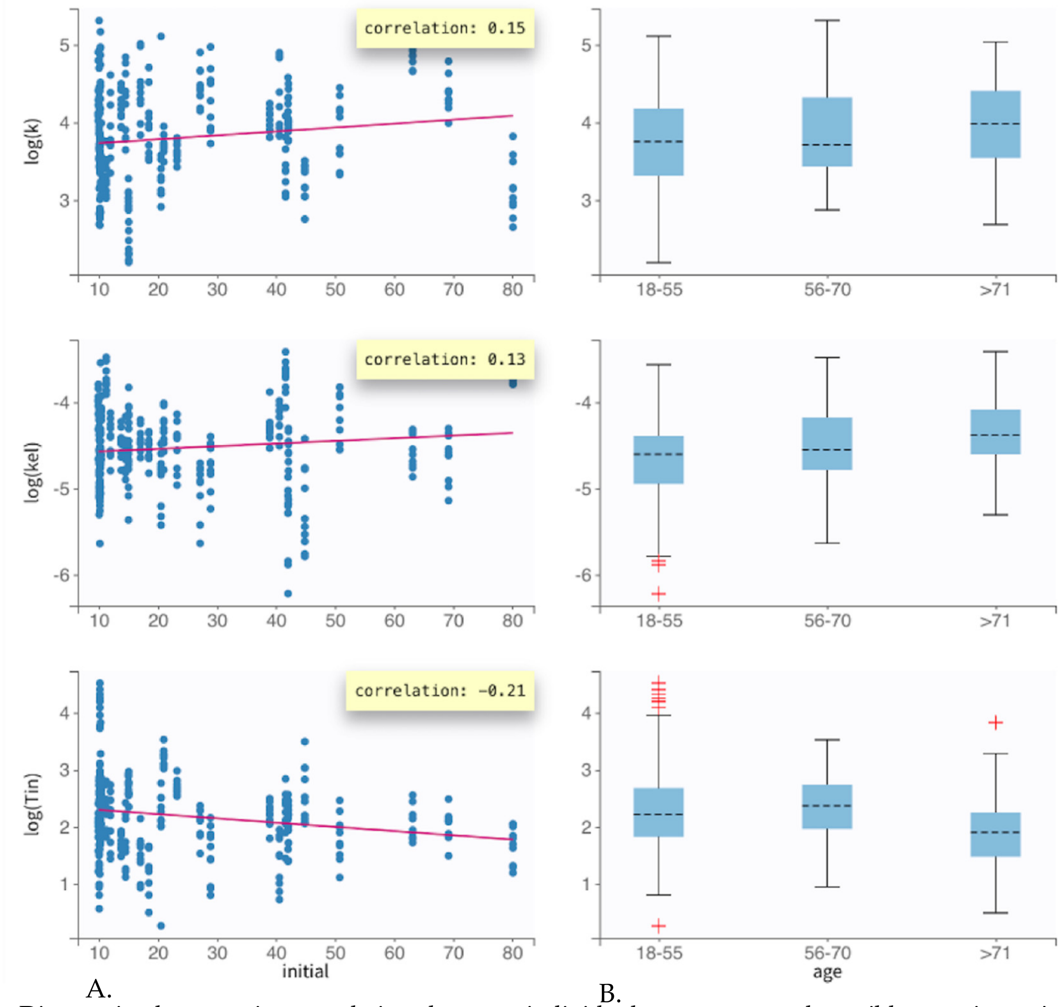

Figure S1. Diagnostic plot assessing correlations between individual parameters and possible covariates. **A.** The initial condition, the nAb titer at day 0, and **B.** age were assessed as potential covariates. No significant correlations were found.

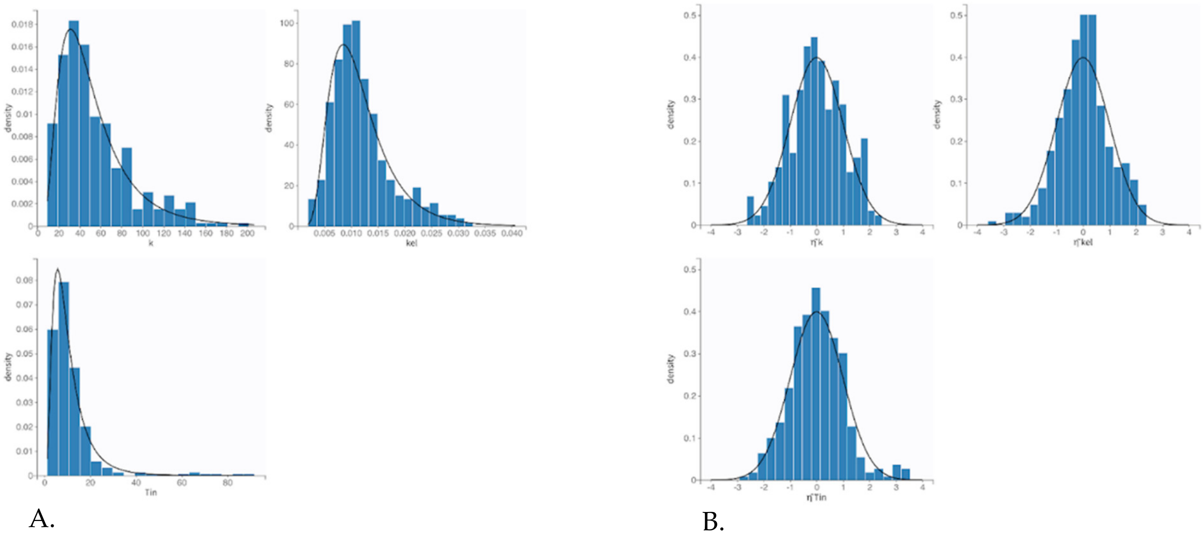

Figure S2: Probability distribution of individual parameter estimates and random effects for the nAb kinetics model. **A.** Probability distribution of individual parameters. The histogram plots represent the empirical distribution. The black line represents the theoretical distribution defined in the statistical model, which is a log-normal distribution for each parameter. **B**. Probability distribution of standardized random effects. The histogram plots represent the empirical distribution. The black line represents the theoretical distribution defined in the statistical model, which is a normal distribution for each random effect.

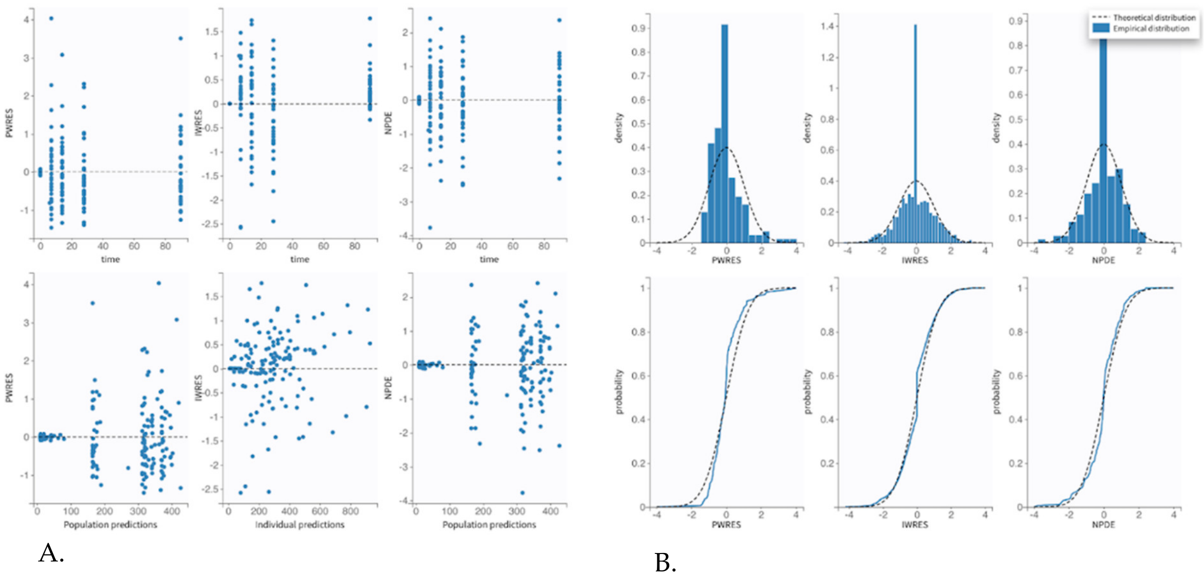

Figure S3. Goodness-of-fit analysis for nAb kinetics model residuals. **A.** Scatter plot of the residuals. These plots display the PWRES (population weighted residuals), the IWRES (individual weighted residuals), and the NPDEs (normalized prediction distribution errors) as scatter plots with respect to time and prediction. Residuals should be randomly scattered around the x-axis, which confirms suitability of the proportional error model. **B.** Distributions of the residuals. Empirical and theoretical probability density function (PDF) of the PWRES, IWRES and NPDE are shown in the top of the panel. Empirical and theoretical cumulative distribution function (CDF) are at the bottom. This normality check confirms suitability of the error model.

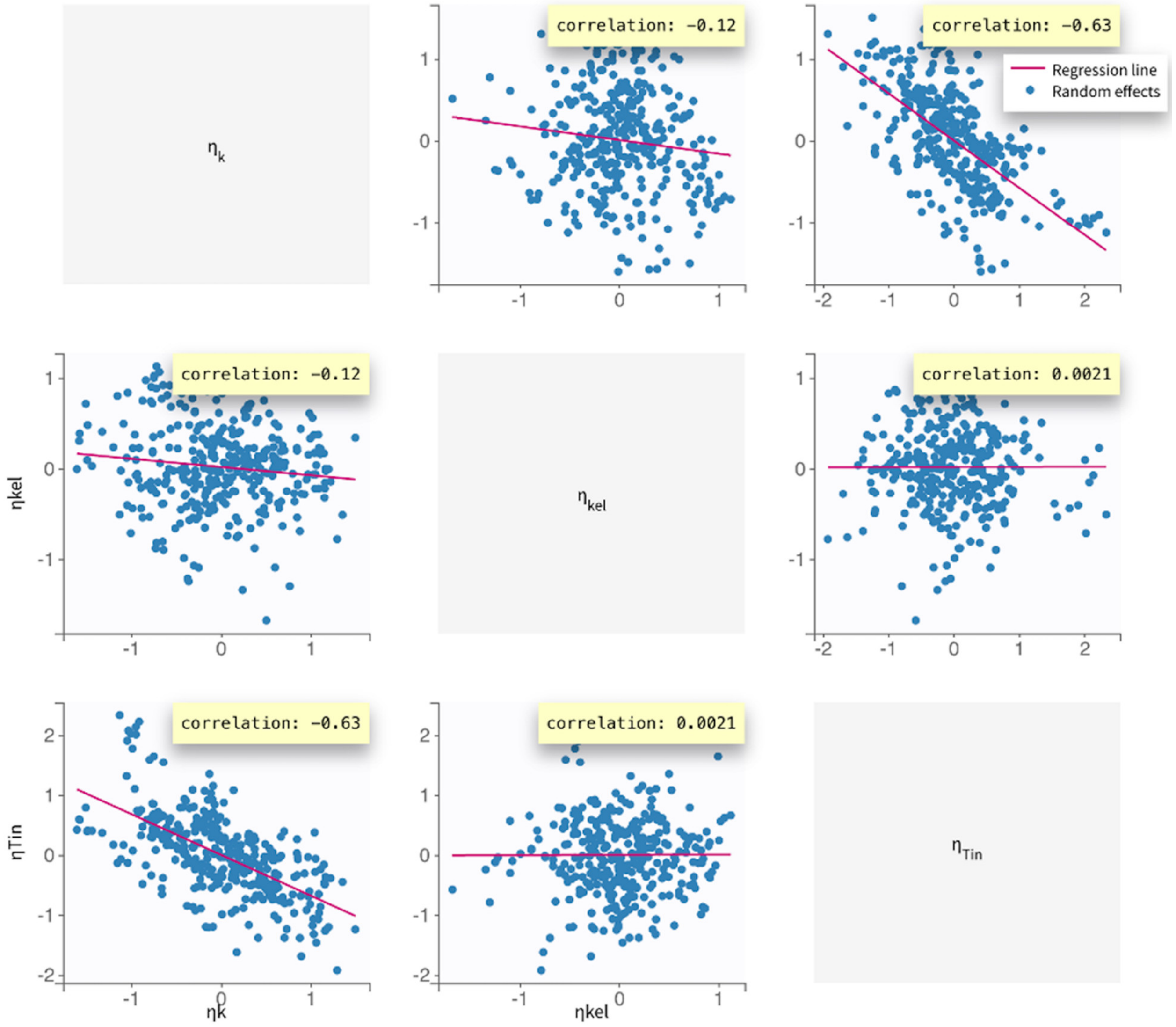

Figure S4. Correlation between parameters in the nAb kinetics model. There is a moderate correlation between Tin and k_p.

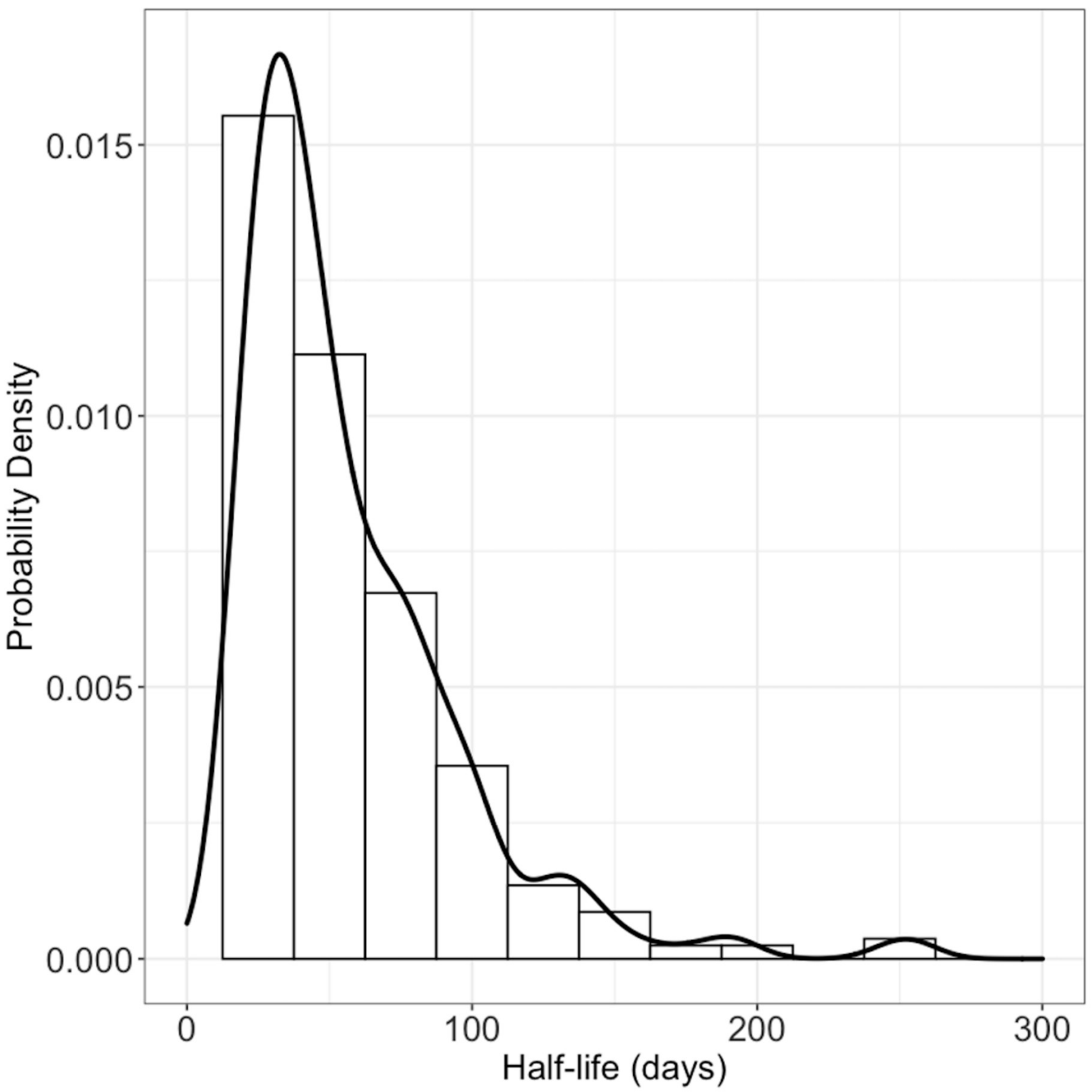

Figure S5: Probability distribution of half-lives for vaccine-induced IgG for individuals in the study population.

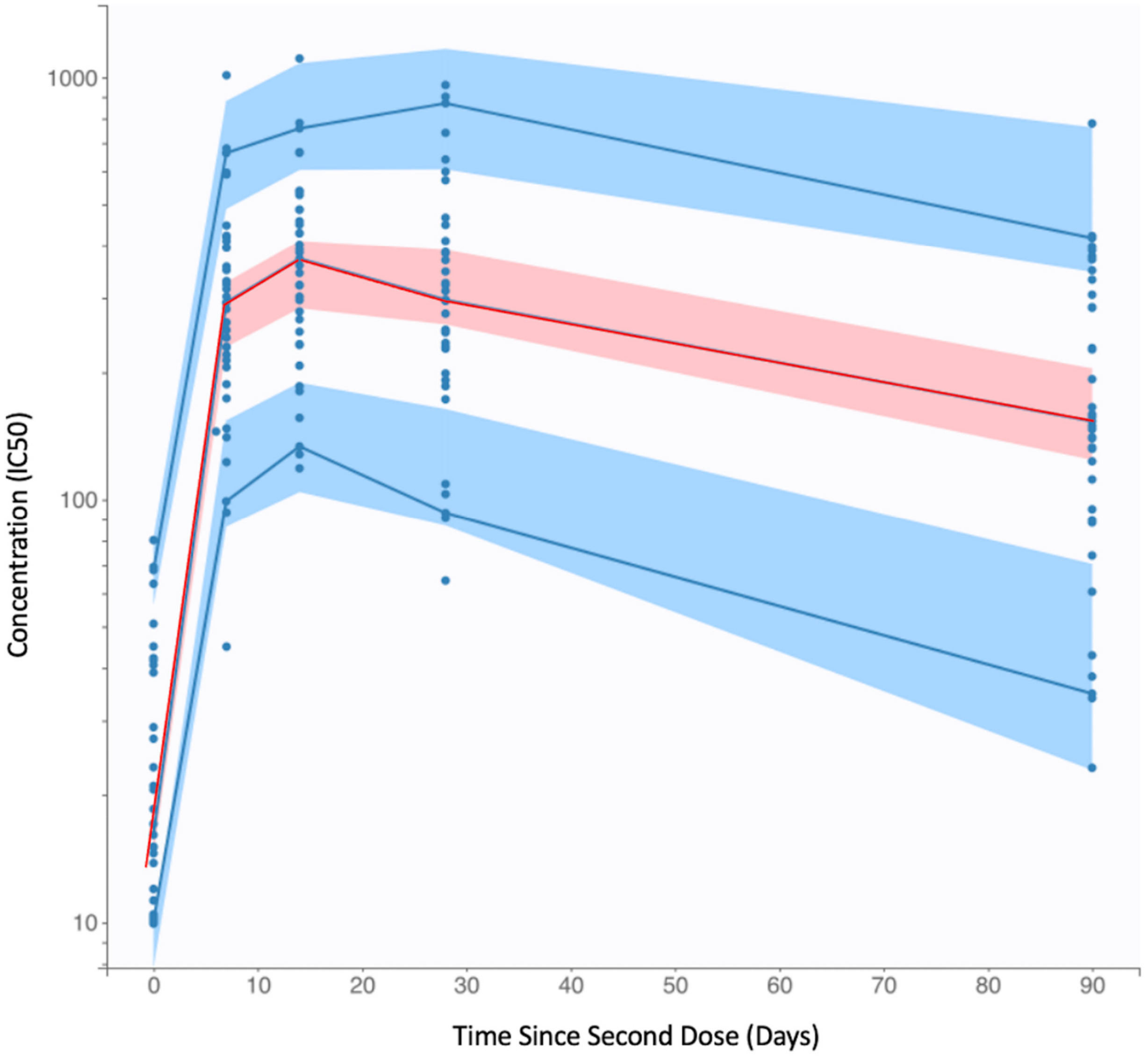

Figure S6. VPC of IgG kinetics model fit with 90% prediction intervals. Blue dots represent published data characterizing SARS-CoV-2 IgG titers following vaccination. The red line represents the empirical 50th percentile and the blue lines represent the empirical 10th and 90th percentiles. The shaded regions represent the model's 90% prediction intervals for the 50th percentile (pink) and 10th and 90th percentiles (blue).

Table S1. Parameter values for fitted IgG kinetics model with standard errors (SE) and relative standard error (RSE).

Parameter	Value	Units	Standard error	Relative standard error (%)
			Fixed effects	
$k_{p,\ pop}$	0.57	1/days	0.053	9.32
$k_{el,\ pop}$	0.012	1/days	0.0025	20.3
$T_{in,\ pop}$	4.54	days	0.61	13.5
			Standard deviation of the random effects	
ω_{kp}	0.15	1/days	0.043	27.8
ω_{kel}	0.28	1/days	0.064	22.8
ω_{Tin}	0.15	days	0.061	39.9
			Correlations	
$corr_{kel,Tin}$	0.9		0.2	22.6
$\beta_{kel,\ age\ 56\text{-}70}$	0.95		0.23	23.9
$\beta_{kel,\ age\ >70}$	0.54		0.2	38.0

$\beta_{kel, initial}$	-0.0000026	0.0000022	83.8
$\beta_{Tin, age 56-70}$	0.52	0.1	20.1
$\beta_{kel, age >70}$	0.29	0.098	33.4
$\beta_{Tin, initial}$	-0.0000038	0.0000062	16.5
	Error model parameters		
**b**	0.3	0.021	6.99

b is the coefficient of proportional error. β coefficients represent the degree of change in the kinetics parameter for every change of covariate variable. .

Table S2. AIC values for prospective model structures.

Object	Model	AIC
**Nabs**	0-order production	1842.04
**Nabs**	1st-order production	1894.46
**IgG**	0-order production	4278.69
**IgG**	1st-order production	4261.57

Table S3. Comparison of model-predicted vaccine efficacy over time with clinical data.

Variant	Time since second dose (days)	VEm, model estimate (%)	VEm, clinical (%, 95% CI) [40]
**Delta**	14-90	76.4	82.8 (69.6, 90.3)
**Delta**	90-180	57.2	63.6 (51.8, 72.5)
**Omicron**	14-90	22.6	30.4 (5.0, 49.0)
**Omicron**	90-180	11.9	15.2 (0.0, 30.7)
